# The Effect of Ejaculatory Abstinence Interval on Sperm Parameters and Clinical Outcome of ART. A Systematic Review of the Literature

**DOI:** 10.3390/jcm10153213

**Published:** 2021-07-21

**Authors:** Piotr Sokol, Panagiotis Drakopoulos, Nikolaos P. Polyzos

**Affiliations:** 1 Department of Reproductive Medicine, Dexeus University Hospital, 08028 Barcelona, Spain; nikpol@dexeus.com; 2 Centre for Reproductive Medicine, Universitair Ziekenhuis Brussel, 1090 Brussels, Belgium; p.drakopoulo@gmail.com; 3 Centre for Reproductive Medicine, Vrije Universiteit Brussel, 1050 Brussels, Belgium; 4 Faculty of Medicine and Health Sciences, University of Ghent (UZ Gent), 9000 Gent, Belgium

**Keywords:** ejaculatory abstinence, semen analysis, sperm parameters, sperm DNA fragmentation, in vitro fertilization, infertility

## Abstract

Since the publication of the first edition of the WHO (World Health Organization) Laboratory Manual for the examination of Human Semen in 1980, the reference values of sperm parameters have been updated on four occasions. Currently and globally, most of the laboratories analyzing semen samples use the latest, 5th edition of the manual that recommends ejaculatory abstinence from two to seven days before producing the sample for examination. While this standardized interval of time facilitates the interpretation of the results and research, no solid evidence exists to support the WHO-recommended abstinence time for a semen analysis in order to optimize clinical outcomes after assisted reproduction. Most of the studies refer to different clinical outcomes, different groups of patients and different editions of the WHO Laboratory Manual, including heterogeneous intervals of abstinence or sperm parameters. The aim of the current systematic review was to evaluate available evidence correlating ejaculatory abstinence time with clinical outcomes and sperm parameters analyzed according to the last edition of the World Health Organization Laboratory Manual reference values in different male populations. The results from the included studies indicate that WHO abstinence recommendations may need revision, given that a shorter ejaculatory abstinence interval appears to be associated with improved sperm parameters, such as sperm DNA fragmentation, progressive motility or morphology, while evidence suggests a potential increase in embryo euploidy rates and pregnancy outcomes.

## 1. Introduction

For the past 40 years of the management of infertility, we have widely adopted recommendations based on the World Health Organization (WHO) Laboratory Manual for the Examination of Human Semen [1,2,3,4,5] to standardize semen analysis and to confirm the presence of male factor. These guidelines set up a universal standard and reference point in the assessment of basic semen parameters worldwide.

It is well known that semen parameters may present an important biological variation [6,7], especially intra-individually [8,9]. It is not a surprise if we consider ejaculate as a final product obtained from testes and male accessory glands and after being released from the genital tract. The abstinence time, ejaculatory frequency, general health status, scrotal conditions, surgery, genital or urinary tract infections, lifestyle or medications are only a few factors that may have an impact on the sperm quality [10,11].

The relatively long physiological duration of spermatogenesis in humans further complicates the assessment of any factor or intervention that could impact spermatogenesis.

Due to the high variation of sperm parameters, one should not expect to obtain a full view of semen quality by analyzing only one semen sample [4], with several groups suggesting analysis of three or more semen samples in an attempt to reduce the intra-individual coefficient of variation for at least some of the studied parameters [11,12].

At present, the formal recommendation regarding abstinence time for semen analysis according to the last edition of the WHO Manual is from two to seven days [4]. Nonetheless, this period is not supported by the European Society of Human Reproduction and Embryology (ESHRE) and Nordic Association for Andrology (NAFA) guidelines [13] that limit abstinence time to a narrower interval, ranging from three to four days. Owing to the abovementioned conflicting evidence regarding the optimal abstinence interval prior to ART [14,15,16,17], over the last three decades a growing number of articles have been published addressing the impact of ejaculatory abstinence period on different sperm parameters or clinical outcomes, with controversial results. Previously published review articles [18,19] supported that the relationship between the abstinence time and sperm quality is not straightforward given that while some parameters (e.g., motile sperm cells) might improve with shorter abstinence time, others (e.g., total sperm count), might do the opposite, whereas a recent meta-analysis [20] suggested that a shorter (less than four days) rather than longer (from four to seven days) abstinence period could result in better clinical outcome in couples undergoing IVF/ICSI.

Taking into account the conflicting results published to date, we set to perform a systematic review of the literature in order to shed more light on the available evidence relating the ejaculatory abstinence period with sperm parameters and clinical outcomes in different male populations and clinical settings.

## 2. Materials and Methods

An electronic search through PubMed/MEDLINE was conducted. We included only articles published in English and after 2010. Publications with both normal and abnormal semen results were included. However, studies reporting outcomes after surgical sperm retrieval were excluded. To select only publications of interest we restricted the search by using the following keywords: “semen sample”, “sperm parameters”, “sexual abstinence”, “ejaculatory abstinence”, “clinical outcome”, “semen parameters”, “semen analysis”, “sperm count”, “sperm motility”, “sperm morphology”, “sperm vitality”, “IVF”, “in vitro fertilization”, “ICSI”, “intracytoplasmic sperm injection”, “IUI”, “intrauterine insemination”, “DNA fragmentation” and “euploidy”.

We included both retrospective and prospective studies. The reference list of the initially selected articles was reviewed to add more relevant studies that were not included in the initial search. To have uniform data, studies using WHO reference values before its 5th edition (prior to 2010) were excluded from the review.

We did not apply any restriction regarding the abstinence time. All the possible settings (short abstinence period—SAP, currently recommended by WHO 5th Edition Manual of a 2-to-7-day abstinence period—RAP, or long abstinence period—LAP) were accepted.

After careful initial assessment and application of filters mentioned before, we accepted 24 publications for this review.

## 3. Results

For easier interpretation of data, we divided the studies into three categories depending on the semen results:

Group (I) with normal results, deriving from sperm donors, healthy volunteers and patients with no apparent male factor;

Group (II) with abnormal results, coming from patients undergoing fertility treatment and

Group (III) with mixed, including both normal and abnormal, results. Studies were placed in this group when it was not possible to place them either in the Group (I) or (II).

In total, 24 publications were included. The most relevant information regarding the incorporated studies is included in Table 1. It contains data regarding the influence of the ejaculatory abstinence period on specific sperm parameters in different populations such as healthy volunteers, donors and infertility patients (listed in the upper and central part of the table). Additionally, the lower part of the table includes studies evaluating the influence of abstinence time on clinical outcomes. Some of the publications were listed in the table more than once since we could identify the results for different populations within the same publication, e.g., we have listed Shen’s work three times, given that the authors presented findings for the influence of the abstinence period on semen parameters of volunteers (I), infertility patients (III) and clinical outcomes.

All the included studies followed the 5th edition of WHO Laboratory Manual reference values when analyzing semen samples. The only exception was the study of Sukprasert et al. [23], where the authors used WHO reference values from 1999. The study included only healthy individuals with normal semen results and therefore the inclusion of this study in the present review did not present a dilemma to accept the results under WHO 2010 reference values. In three other studies [21,37,42] we could not assess whether the most recent or the former WHO reference values were used. Nevertheless, data from these publications did not hamper the overall concept of having as uniform information as possible.

We included 15 studies in the category of normal semen results (I); two of which were represented by sperm donors (112 males, 224 samples), eight by healthy volunteers (182 males, 563 samples) and six by patients attending fertility units (1903 males, 2065 samples). Thirteen studies were prospective observational, one was RCT and one was a retrospective study.

In the group with abnormal semen results (II) which included 1408 males, 1632 samples were included, and data were collected from eight studies. Five studies were prospective observational and three were retrospective.

In the mixed group (III) we analyzed results from three studies [27,39,40] that included 3355 males who produced 3522 samples. Two studies were prospective and one retrospective.

Finally, five studies (three prospective observational and two retrospective) reported the outcome of the IVF/ICSI cycle with the use of own or donated eggs. In total, 2680 patients were included, of which 45 underwent ovum donation cycle [37].

Only one study reported the outcome of intrauterine insemination (IUI) [30].

We registered the relationship between different ejaculatory abstinence periods and the following sperm parameters: volume, total sperm count, progressive motility, morphology, vitality and sperm deoxyribonucleic acid (DNA) fragmentation. We additionally reviewed clinical outcomes after IVF / ICSI cycles, IUI and embryo aneuploidy rate depending on the ejaculatory abstinence time.

The studies included in this review were conducted in Asia, North and South America, Europe and Africa. The largest studies were conducted in Brazil and India and included four thousand cases in total. The median age of the participants was between 30 and 40 years in the majority of studies and ranged from 20 to 65 years old.

The abstinence time reported in the studies varied from less than a day (in 12 studies, with as short a time as 40 min [38]) to as long as 30 days or more [33]. The majority of studies (18 out of 24) compared the effect of short abstinence period (SAP; <2 days) with the WHO-recommended abstinence period (RAP; within 2 to 7 days) on sperm parameters or clinical outcome. Four studies did not restrict the abstinence time to WHO recommendations [17,33,35,40]. Two studies examined possible effects of long abstinence period (LAP) [41,42] on the clinical outcome.

### 3.1. Semen Volume

(I), (II), (III)—21 out of 24 studies analyzed the relationship between ejaculate volume and the abstinence time. All the studies reported a positive correlation between the abstinence time and semen volume when SAP was compared with RAP and RAP with LAP.

### 3.2. Total Sperm Count

Total sperm count was evaluated in 12 studies.

(I), (II), (III)—Results were consistent among all the studies within all the groups. All studies (2063 males, 2560 samples) except for one in the Group (II) (25 males, 75 samples) [36] indicated that total sperm count was lower when abstinence time was shorter when comparing SAP with RAP. The same results were obtained when RAP was compared with LAP when assessed in volunteers [17] and patients with abnormal semen sample [35].

### 3.3. Progressive Motility (a + b)

Eleven studies in Group (I), eight in group (II) and five in group (III) analyzed the effect of abstinence on progressive motility.

(I)—According to eight studies (1806 males, 2033 samples), the duration of abstinence time did not show any impact on progressive motility when SAP with RAP and RAP with LAP were compared. However, in four studies (261 males, 522 samples) SAP was related to an increased fraction of progressive motile spermatozoa when compared with RAP.

(II)—Four studies (1231 males, 1253 samples) reported no influence of the abstinence time on progressive motility, while the other four studies (177 males, 379 samples) indicated that SAP was related with higher progressive motility when compared with RAP. The results were similar when RAP was compared with LAP.

(III)—Conflicting data were noted in this group. While two studies (2149 males, 2261 samples) showed no impact of different abstinence time on progressive motility, two different studies (2625 males, 2792 samples) showed higher progressive motility rate and one study (730 males and samples) showed lower progressive motility rate in men with shorter abstinence interval.

### 3.4. Sperm Morphology

Sperm morphology was evaluated in 11 studies in group (I), five studies in group (II) and four studies in group (III).

(I)—Eight studies (1826 males, 2053 samples) did not find any difference between different abstinence periods and sperm morphology. Manna et al. 2020 (30 males, 60 samples) noted that SAP favored a higher rate of normal sperm morphology in normozoospermic patients. Similar results were registered by Shen et al. in healthy volunteers (20 males, 40 samples) [27]. On the contrary, Sukprasert et al. (57 males, 171 samples) [23] noted a lower rate of normal sperm morphology after SAP in males with normal semen result. It is worth mentioning that in the latter study, despite lower values of normal morphology in the SAP group, the results remained within the normal range. If only samples from normozoospermic patients were considered, then SAP would result in similar or better morphology when compared with RAP.

(II)—Three studies (1210 males, 1232 samples) did not find any difference between different abstinence time and sperm morphology. Two studies (108 males, 216 samples) noted that SAP favored a higher rate of sperm cells with normal morphology when compared to RAP. The results were similar when RAP was compared with LAP.

(III)—Three publications (5225 males and samples) did not find any difference between different abstinence time and sperm morphology in all spectrums of abstinence time (SAP, different duration time within RAP, LAP). One study (65 males, 130 samples) noted improved normal morphology rate in the group of patients with SAP when compared to RAP.

### 3.5. Vitality

Sperm vitality was recorded in seven studies.

(I)—In the population of healthy donors or volunteers, ejaculatory abstinence time did not have an impact on the vitality status of spermatozoa according to four studies (132 males, 360 samples). The same sperm vitality was noted across short, standard and long ejaculatory abstinence periods. However, according to one study, SAP resulted in a higher rate of sperm vitality (20 males, 40 samples) [27]. There were no publications regarding sperm vitality in patients with a normal semen sample.

(II)—Sperm vitality was not registered in this group.

(III)—According to Sunanda et al. [39], within the WHO-recommended abstinence period, sperm vitality decreased with the increase of abstinence time (2–3 days vs. 4–5 days vs. 6–7 days). Comar et al. noted similar results when SAP was compared with RAP [40]. Additionally, the group noted that abstinence time longer than five days negatively impacted sperm vitality.

### 3.6. Sperm DNA Fragmentation

Sperm DNA fragmentation was recorded in 16 studies, 12 in group (I), 4 in group (II) and 2 in group (III).

(I)—Nine studies (284 males, 691 samples) found lower sperm DNA fragmentation after SAP when compared with RAP. Similar results were obtained when RAP was compared with LAP. However, in the latter case, there was a scarce presence of results corresponding to LAP (>7 days). There were only 14 samples from seven volunteers registered in the group of Agarwal [17].

Five studies (249 males, 454 samples) did not show different DNA fragmentation rates between SAP and RAP groups.

(II)—Homogeneous results were noted in this group. Shorter abstinence time resulted in lower DNA fragmentation according to four studies (872 males, 951 samples). Three of them compared SAP (<1 day) with RAP. In one study there were no restrictions applied regarding the abstinence time.

(III)—Uniform results were obtained in this group. Shorter abstinence time resulted in lower DNA fragmentation according to two studies (2625 males, 2792 samples). One study compared SAP (<1 day) with RAP, while the other one did not restrict the abstinence time.

### 3.7. Embryo Euploidy

Embryo euploidy rate was assessed in only one study, published by Scarselli et al. [34]. The group found a higher rate of euploid embryos in patients with SAP when compared with RAP. The rate of mosaic embryos was similar between the two groups. There was no information regarding clinical outcomes.

### 3.8. IVF/ICSI

Five studies compared clinical outcomes from IVF/ICSI cycles in patients with different abstinence time.

Two prospective studies (718 cycles) compared SAP (<1 day) with RAP. In both studies, a higher clinical pregnancy rate was noted in patients from the SAP group. Additionally, Shen et al. noted higher live birth rate in the SAP group when frozen embryo transfer cycles were compared.

Two retrospective studies compared the outcomes between the RAP (2–7 days) and the LAP group (>7 days). Lee et al. (449 cycles) did not find any difference regarding implantation, clinical and ongoing pregnancy rates between the groups [41], while Periyasami et al. (1030 cycles) found higher clinical pregnancy and live birth rates in the RAP group [42].

Finally, Borges et al. (483 cycles) did not apply any restriction regarding the abstinence period and compared the outcomes between the group with abstinence time <4 days and >4 days. The group found that shorter abstinence time favored higher implantation and clinical pregnancy rates [35].

### 3.9. IUI

One randomized controlled trial [30] compared IUI outcome between the SAP and RAP groups. In total, 120 patients were included in the study and the clinical pregnancy rate did not differ between the groups.

The comparison of the most relevant results between the group of patients with normal and abnormal semen parameters is presented in Table 2. It shows which abstinence period (SAP vs. RAP, RAP vs. LAP) is more favorable for each sperm parameter. Additionally, it highlights the abstinence interval with better clinical outcome.

## 4. Discussion

In the present review, we evaluated the association between the ejaculatory abstinence interval with clinical outcomes and semen parameters. According to the pooled data, the most relevant functional parameters such as progressive motility, morphology or sperm DNA fragmentation are likely to improve after shorter ejaculatory abstinence in males with both normal and abnormal semen analysis.

The influence of the ejaculatory abstinence period on sperm parameters was recently assessed in two systematic reviews; albeit the available data were highly heterogeneous [18,19]. None of the investigators precisely evaluated the abstinence time or male fertility status and included numerous studies using reference values for semen analysis from the 5th, 4th and even earlier editions of WHO manual. According to both reviews, the longer abstinence time was associated with higher seminal volume, total sperm count [18] and concentration [19]. Interestingly, the abstinence time exceeding five [18] or even seven days [19] was associated with the most relevant increase of sperm count. Thus, these findings would imply the recommendation of longer rather than shorter abstinence time before ejaculation for ART.

Nonetheless, the results regarding the rest of the sperm parameters, i.e., sperm motility, morphology and DNA fragmentation, are more contradictory and inconclusive. While progressive motility could improve with shorter abstinence time according to some [19], others [18] found the results that were very heterogeneous to allow safe conclusions in favor or against long or short abstinence intervals. In contrast to the previously mentioned systematic reviews, the pooled data in the present work shows consistent benefit of SAP regarding the functional sperm parameters, such as progressive motility, morphology or sperm DNA fragmentation. A potential explanation for this discrepancy could be the inclusion of different studies compared with previous systemic reviews, as well as the stricter inclusion criteria utilized in the present review, allowing us to perform separate analysis for the groups with normal/abnormal semen results and different abstinence intervals.

Similarly, the clinical outcomes following IVF/ICSI treatment appear to be associated with the length of ejaculatory abstinence. The available evidence presented in the current review suggests that shortening the abstinence period may result in better clinical pregnancy and live birth rates. Two studies that specifically compared SAP with RAP reported that the clinical pregnancy rates were higher after short abstinence [27,37]. Notably, Sánchez-Martin et al. [37] included only a small number of patients undergoing IVF/ICSI cycle with either autologous or donated oocytes and did not report LBR. Shen et al. [27], on the other hand, while failing to demonstrate a clear benefit of short abstinence in fresh cycle live birth rates, did demonstrate significantly higher cumulative LBR following subsequent FET cycles. The benefit should be therefore expected, at least in terms of improved clinical pregnancy rate, in the SAP group when compared with RAP and similar or better in RAP when compared with LAP. Additionally, LBR could be expected to be higher after RAP rather than LAP when fresh cycles are performed, according to Periyasami et al. [42]. Li et al., who recently published a meta-analysis on the topic, noted similar results regarding clinical pregnancy rates [20]. However, the authors used a different strategy by establishing a cutoff value for the abstinence period of four days and therefore compared the outcomes between the patients who abstained for less than four days or between four and seven days. In comparison to Li´s work, our pooled data indicated better CPR and possibly better LBR after FET cycles when SAP was compared to RAP. Furthermore, after comparing RAP with LAP, we noted that the latter could impair the clinical outcomes.

An interesting finding of our systematic review is that although current evidence suggests that rather shorter abstinence time improves clinical outcome, the same cannot be stated for all the sperm parameters, given that while some of them improve with SAP, others do not. A relevant question is why higher clinical pregnancy and live birth rates go in parallel with improved sperm progressive motility and/or DNA fragmentation after SAP, rather than with higher semen volume and/or total sperm count after RAP or LAP. Is it the functional rather than numeric aspect of the semen sample and sperm cells that plays a major role in the process of fertilization and the development of the viable embryo? A potential explanation could be that human spermatozoa should not be stored for a long period of time in reproductive ducts, since this may have a potentially detrimental effect on sperm quality associated with oxidative stress related to sperm storage. At the same time, SAP could improve sperm protein expression responsible for its functionality, e.g., motility, capacitation or acrosome reaction [27].

Unfortunately, we could only include one study evaluating ejaculatory abstinence time and the outcome of the IUI treatment [30] and this did not demonstrate any benefit for any particular abstinence time (one day vs. three days). The previously mentioned systematic reviews highlighted higher pregnancy rates after IUI when the semen sample was produced after less than two [19] or three days [18].

The strength of this systematic review is the meticulous review of the literature to capture all the trials that assessed sperm parameters according to the reference values from the 5th edition of the WHO Manual. This limited the presence of heterogeneous results, especially among patients with abnormal semen results. Furthermore, the comprehensive evaluation of all available studies including different abstinence intervals and populations allows for better understanding of the different clinical scenarios.

Nevertheless, the current review has also several limitations that merit attention. The inclusion of small, as well as retrospective studies, might have increased the risk of selection bias. Additionally, some of the included studies did not evaluate all routine sperm parameters, which may limit the external validity of the results. The aforementioned drawbacks, along with the clinical heterogeneity of the included studies, prevented us from conducting a meta-analysis. The high intra-individual biological variation of semen parameters should also be taken into account as it may affect the findings.

Lastly, it is essential to highlight that the ejaculatory abstinence period is just one of the several factors contributing to the final semen specimen and fertilization of the oocyte. While analyzing semen quality or counseling couples regarding the impact of male contribution on natural fertility or assisted reproduction, one should always bear in mind often forgotten or underestimated factors such as general health status [10], male age [43], body mass index [44] or socio-psycho-behavioral [45] factors. Furthermore, the duration of sexual arousal before masturbation [46], specific sexual stimuli [47] or obtaining ejaculate via intercourse [48] could also modulate semen and sperm quality.

In conclusion, a shorter than routinely recommended at present (up to seven days based on the WHO Manual) ejaculatory abstinence interval may result in improved sperm parameters, such as sperm DNA fragmentation, progressive motility or morphology with a potential clinical benefit in IVF/ICSI cycles. In this regard, patients should be discouraged from abstaining for a prolonged time due to the potential negative impact on both semen parameters and clinical outcomes. Further studies to better define the optimum abstinence interval within the SAP are urgently warranted.

## Figures and Tables

**Table 1:** Details from included studies regarding ejaculatory abstinence period, semen parameters and clinical outcome.

Publication		WHO Manual Edition	Type of Study	Population	ABSTINENCE in Days (hours)	Volume	Total Sperm Count	Motility (a + b)	Morphology	Vitality	DNA Frag.	Aneuploidy	IUI	IVF/ICSI		
Gosalvez et al. [21]	2011		PROSPECTIVE	donors, *n* = 12 (24)	<1 (3 h) vs. 4						/\				NORMOZOOSPERMIA	SEMEN RESULTS
Ayad et al. [22]	2017	5th	PROSPECTIVE	donors, *n* = 100 (200)	<1 (4 h) vs. 4	/\	/\	\/	<>	<>	<>					
Sukprasert et al. [23]	2013	4th	PROSPECTIVE	volunteers, *n* = 57 (171)	2–5 vs. <1 (18–30 h) vs. 4	/\			/\		/\					
Okada et al. [24]	2020	5th	PROSPECTIVE	volunteers, *n* = 50 (100)	1 vs. 4	/\	/\	\/	<>		/\					
Agarwal et al. [17]	2016	5th	PROSPECTIVE	volunteers, *n* = 7 (42)	1; 2; 5; 7; 9; 11	/\	/\	<> (a + b + c)	<>	<>	/\					
Mayorga-Torres et al. [16]	2015	5th	PROSPECTIVE	volunteers, *n* = 6 (42)	1 vs. 3–4	/\	/\	<>	<>	<>	<>					
Mayorga-Torres et al. [25]	2016	5th	PROSPECTIVE	volunteers, *n* = 3 (12)	<1 (2 h–4 h–6 h) vs. 3–4	/\ (6 h)	/\	<>			<>					
Uppangala et al. [26]	2016	5th	PROSPECTIVE	volunteers, *n* = 19 (76)	1 vs. 3 vs. 5 vs. 7	/\	/\	<>	<>	<>	/\ (5; 7)					
Shen et al. [27]	2019	5th	PROSPECTIVE	volunteers, *n* = 20 (40)	<1 (1–3 h) vs. 3–7				\/	\/	/\					
Welliver et al. [28]	2016	5th	PROSPECTIVE	volunteers, *n* = 20 (80)	1 vs. 3–5	/\		<>	<>		<>					
Alipour et al. [29]	2017	5th	PROSPECTIVE	patients, *n* = 43 (86)	<1 (2 h) vs. 4–7	/\	/\	\/								
Gosalvez et al. [21]	2011		PROSPECTIVE	patients, *n* = 21 (42)	1 vs. 4						/\					
Kabukçu et al. [30]	2021	5th	RCT	patients, *n* = 60 + 60	1 vs. 3	/\	/\	<>	<>		<>					
Dahan et al. [31]	2021	5th	PROSPECTIVE	patients, *n* = 68 (136)	<1 (3 h) vs. 3	/\		\/			/\					
Manna et al. [32]	2020	5th	PROSPECTIVE	patients, *n* = 30 (60)	<1 (1 h) vs. 2–7	/\		<>	\/		/\					
Dupesh et al. [33]	2020	5th	RETROSPECTIVE	patients, *n* = 1621	<1; 1–2; 3–7; 8–15; 16–30; >30	/\		<>	<>							
Dahan et al. [31]	2021	5th	PROSPECTIVE	patients, *n* = 44 (88)	<1 (3 h) vs. 3	/\		\/			/\				ABNORMAL	
Manna et al. [32]	2020	5th	PROSPECTIVE	patients, *n* = 35 (70)	<1 (1 h) vs. 2–7	/\		\/	\/		/\					
Dupesh et al. [33]	2020	5th	RETROSPECTIVE	patients, *n* = 416	<1; 1–2; 3–7; 8–15; 16–30; >30	/\		<>	<>							
Scarselli et al. [34]	2019	5th	PROSPECTIVE	patients, *n* = 22 (44)	<1 (1 h) vs. 2–5	/\		<>	<>			/\				
Borges et al. [35]	2019	5th	PROSPECTIVE	patients, *n* = 818 (772 abnormal)	no restrictions	/\	/\	<>	<>		/\					
Said and Reed [36]	2015	5th	RETROSPECTIVE	patients, *n* = 25 (75)	<1 vs. 3–4	/\	\/	\/								
Sanchez-Martin et al. [37]	2013		PROSPECTIVE	patients, *n* = 21	<1 (12 h) vs. 4	/\		<>			/\					
Bahadur et al. [38]	2015	5th	RETROSPECTIVE	patients, *n* = 73 (146)	<1 (0.7 h) vs. 2–7	/\		\/	\/							
Sunanda et al. [39]	2014	5th	RETROSPECTIVE	patients, *n* = 730	2–3 vs. 4–5 vs. 6–7	/\	/\	/\	<>	\/					MIXED	
Dahan et al. [31]	2021	5th	PROSPECTIVE	patients, *n* = 112 (224)	<1 (3 h) vs. 3	/\		<>			/\					
Shen et al. [27]	2019	5th	PROSPECTIVE	patients, *n* = 167 (334)	<1 (1–3 h) vs. 3–7	/\	/\	\/								
Comar et al. [40]	2017	5th	PROSPECTIVE	patients, *n* = 2458	no restrictions (<2; 2–5; >5)	/\		\/	<>	\/	/\					
Manna et al. [32]	2020	5th	PROSPECTIVE	patients, *n* = 65 (130)	<1 (1 h) vs. 2–7	/\			\/							
Dupesh et al. [33]	2020	5th	RETROSPECTIVE	patients, *n* = 2037	<1; 1–2; 3–7; 8–15; 16–30; >30	/\		<>	<>							
Kabukçu et al. [30]	2021	5th	RCT	patients, *n* = 60 + 60	1 vs. 3								<>		OUTCOME	
Lee et al. [41]	2018	5th	RETROSPECTIVE	patients, *n* = 449	2–7 vs. >7											
Sanchez-Martin et al. [37]	2013		PROSPECTIVE	patients, *n* = 40 + 150	<1 (12 h) vs. 3–4									<> (CP)		
Periyasami et al. [42]	2017		RETROSPECTIVE	patients, *n* = 1030	2–7 vs. >7									\/ (CP)		
Borges et al. [35]	2019	5th	PROSPECTIVE	patients, *n* = 483	no restrictions (<4 vs. >4)	/\	/\	<>	<>		/\			\/ (CP; LBR)		
Shen et al. [27]	2019	5th	PROSPECTIVE	patients, *n* = 361 + 167	<1 (1–3 h) vs. 3–7									\/ (IR; CP)		
														\/ (CP; LBR in FET)		

/\ means increase with abstinence time; \/ means decrease with abstinence time; <> means no change; blank cell means that the parameter was not registered in the study; RCT—Randomized Controlled Trial; CP—Clinical Pregnancy; IR—Implantation Rate; LBR—Live Birth Rate; FET—Frozen Embryo Transfer cycle.

**Table 2:** Comparison of sperm parameters and clinical outcomes after different abstinence times in patients.

	Sperm Parameter	GROUP		
		I (*)	II	Clinical Outcome
SAP vs. RAP	volume	RAP	RAP	-
	total sperm count	RAP		-
	progressive motility	SAP	SAP	-
	morphology	SAP	SAP	-
	DNA fragmentation	SAP	SAP	-
	embryo euploidy rate	-	SAP	-
	clinical pregnancy rate	-	-	SAP
	live birth rate in FET	-	-	SAP
RAP vs. LAP	volume	LAP	LAP	-
	total sperm count	-	LAP	-
	progressive motility			-
	morphology			-
	DNA fragmentation	-	RAP	-
	clinical pregnancy rate	-	-	RAP
	live birth rate	-	-	RAP
	Parameter better for the indicated abstinence period (SAP/RAP/LAP)			
	Parameter similar or better for the indicated abstinence period (SAP/RAP/LAP)			
	Parameter similar between compared abstinence periods			
	Conflicting data			

* Only patients with normal semen sample (donors and volunteers) were excluded; SAP—short abstinence period; RAP—WHO-recommended abstinence period; LAP—long abstinence period.

## Data Availability

Not applicable.
